# *TET2*-mutant clonal hematopoiesis is associated with peripheral bypass graft occlusion: A multicenter retrospective cohort pilot study

**DOI:** 10.1016/j.jvssci.2026.100442

**Published:** 2026-08-23

**Authors:** Elena Streck, Manuela Harloff, Dennis Freuer, Sebastian Dintner, Dimitrios Dedegkikas, Dominik Jost, Elisabeth Kling, Bruno Märkl, Martin Trepel, Alexander Hyhlik-Dürr, Rainer Claus

**Affiliations:** a Department of Vascular Surgery, Faculty of Medicine, University of Augsburg, Augsburg, Germany; b Department of Pathology, Faculty of Medicine, University of Augsburg, Augsburg, Germany; c Institute of Epidemiology, Faculty of Medicine, University of Augsburg, Augsburg, Germany; d Department of Vascular Surgery, Faculty of Medicine, University of Augsburg, Augsburg, Germany; e Department of Vascular Surgery, Endovascular Surgery, and Vascular Medicine, Elisabethen-Klinikum, Ravensburg, Germany; f Institute for Laboratory Medicine and Microbiology, Faculty of Medicine, University of Augsburg, Augsburg, Germany; g Department of Hematology and Oncology, Faculty of Medicine, University of Augsburg, Augsburg, Germany; h Comprehensive Cancer Center Augsburg (CCCA), University Medical Center Augsburg, Augsburg, Germany

**Keywords:** Clonal hematopoiesis, CHIP, *TET2*, Peripheral arterial disease, Peripheral vascular bypass occlusion, Graft patency

## Abstract

**Objective:**

Clonal hematopoiesis of indeterminate potential (CHIP), defined by somatic mutations in hematopoietic cells, is associated with cardiovascular disease through proinflammatory mechanisms. We investigated whether CHIP, and specifically *TET2* mutations, is associated with peripheral bypass graft occlusion in patients with peripheral arterial disease.

**Methods:**

In this multicenter, retrospective cohort pilot study, 47 patients with peripheral arterial disease who had undergone femoropopliteal or femorocrural bypass grafting were analyzed. CHIP status was assessed from peripheral blood using a 40-gene myeloid sequencing panel. Time-to-event analyses were performed using Kaplan-Meier survival analysis and multivariable Cox proportional hazards regression adjusted for diabetes and smoking status.

**Results:**

Of 47 patients, 33 had bypass graft occlusion and 14 had patent grafts. CHIP was detected in 14 individuals (29.8%). The mutational spectrum differed markedly between groups: in the patent graft group, all CHIP mutations were in *DNMT3A*, whereas *TET2* was the most frequently mutated gene in the occlusion group (7 of 17 mutations; 41%). All five *TET2*-mutant patients belonged to the occlusion group. Cell sorting revealed CHIP variants predominantly in myeloid cells, natural killer cells, and B lymphocytes, with near-absence in T cells. Cox regression demonstrated a significant association between *TET2* mutation and bypass occlusion (hazard ratio, 6.70; 95% confidence interval, 1.96-22.96; *P* = .003).

**Conclusions:**

*TET2* mutations are associated with peripheral bypass graft occlusion and may represent a novel biomarker for graft failure risk. These findings require validation in larger prospective studies.

**Clinical relevance:**

Clonal hematopoiesis, particularly *TET2* mutations, is increasingly recognized as a driver of atherosclerotic cardiovascular disease and peripheral arterial disease (PAD). This pilot study extends that risk to the surgical setting, demonstrating a strong association between *TET2* mutations and peripheral bypass graft occlusion (hazard ratio, 6.70). If confirmed in larger prospective cohorts, preoperative *TET2* screening could identify a high-risk subgroup of patients with PAD requiring intensified postoperative imaging surveillance, more aggressive antithrombotic regimens, earlier reintervention thresholds, and consideration of targeted anti-inflammatory prophylaxis. Such gene-specific risk stratification could shift bypass care toward individualized, biomarker-guided management of patients with PAD undergoing revascularization.

**Article Highlights**

• **Type of Research:** Human study
• **Key Findings:** In 47 patients with peripheral arterial disease and prior femoropopliteal or femorocrural bypass (33 occluded and 14 patent), clonal hematopoiesis of indeterminate potential was detected in 29.8%. All five *TET2*-mutant patients had graft occlusion. *TET2* mutation was associated with occlusion (hazard ratio, 6.70; 95% confidence interval, 1.96-22.96; *P* = .003), persisting after adjustment for the peripheral arterial disease stage.
• **Take Home Message:** *TET2*-mutant clonal hematopoiesis is associated with peripheral bypass graft occlusion and may identify patients at increased risk of graft failure, supporting prospective validation with clonal hematopoiesis of indeterminate potential assessment at the time of bypass surgery.

Peripheral artery disease (PAD) is a chronic condition caused by atherosclerotic plaques, leading to reduced arterial blood flow in the lower extremities. Clinical presentations range from intermittent claudication (Fontaine stage II) to chronic limb-threatening ischemia (Fontaine stages III-IV). Progression of PAD is influenced by known risk factors such as smoking, diabetes, dyslipidemia, hypertension, and inflammation.^1^ Current therapies for patients with symptomatic PAD include percutaneous endovascular interventions, open revascularization, or a combination of both. Despite advances in vascular grafts, surgical technique, and antithrombotic therapy, late graft occlusions (>30 days after surgery) remain frequent, affecting 20% to 50% of patients.^2^, ^3^, ^4^, ^5^ Long-term data show that autologous grafts of the great saphenous vein are superior in terms of primary patency,^6^, ^7^, ^8^ but synthetic femoropopliteal bypasses occlude in 25% to 35% of cases within 2 years.^7^ Within 5 years, approximately half of infrainguinal bypasses fail, and 20% to 30% of patients lose limbs.^9^ Known predictors of graft occlusion include graft type, previous vascular interventions, concomitant cardiovascular disease, advanced PAD stage, intimal hyperplasia, and nonuse of anticoagulants.^7^ Although elevated inflammatory biomarkers have been associated with adverse outcomes, clinically useful biomarkers that reliably identify patients at increased risk of bypass graft occlusion remain lacking.

Recent evidence links clonal hematopoiesis of indeterminate potential (CHIP)—characterized by somatic mutations in hematopoietic cells without overt hematologic malignancy—to increased all-cause mortality and cardiovascular events, including atherosclerosis and myocardial infarction.^10^, ^11^, ^12^ The prevalence of CHIP increases with age, affecting more than 10% of individuals older than 70 years, with mutations in the epigenetic regulators *DNMT3A*, *TET2*, and *ASXL1* being most common.^13^

The link between CHIP, inflammation, and cardiovascular disease is supported by large epidemiological studies and by mechanistic mouse models demonstrating a causal contribution of *TET2* deficiency to atherosclerosis. CHIP carriers have a 1.7- to 2.0-fold increased risk of coronary artery disease and premature myocardial infarction.^11^ Although the association between CHIP and atherosclerotic cardiovascular disease is now well established, its impact on the outcomes of vascular surgical interventions has not been investigated. Specifically, whether CHIP influences peripheral bypass graft patency remains unknown.

We hypothesized that CHIP is associated with an inflammatory milieu that predisposes patients to bypass graft occlusion and that TET2 mutations, given their established role in macrophage-mediated inflammation, are particularly associated with graft failure. To test this hypothesis, we performed a multicenter, retrospective cohort pilot study comparing the prevalence of CHIP mutations in patients with PAD with and without peripheral bypass graft occlusion.

## Patients and methods

### Study population and procedures

We performed a multicenter, retrospective cohort pilot study at three centers (University Medical Center Augsburg, Klinik Günzburg, and St. Elisabethen-Klinikum Ravensburg) from July 2020 to April 2022. Adult patients with a history of PAD who had undergone open surgical bypass (femoropopliteal or femorocrural) and were receiving antiplatelet or anticoagulant therapy were eligible. Patients were screened consecutively at each center; no additional selection beyond the eligibility criteria was applied. Inclusion criteria required a diagnosis of PAD, prior bypass surgery, age ≥18 years, and ability to provide informed consent.

Patency or occlusion was assessed by computed tomography angiography, digital subtraction angiography, or duplex ultrasound imaging. Exclusion criteria were autoimmune diseases, malignant hematological diseases, bypass occlusion for technical reasons (eg, graft kinking and anastomotic defect within 30 days), and active infections. Full eligibility criteria are provided in Supplementary Table I (online only). Baseline data on demographics, comorbidities, laboratory values, PAD stage (Fontaine classification), and bypass graft characteristics were obtained from medical records. All subjects gave written informed consent. Peripheral blood was collected once at study inclusion, either at the time of diagnosis of bypass graft occlusion or, in patients with patent grafts, during routine follow-up. CHIP mutations were assessed using peripheral blood (PB) samples collected at the time of enrollment, after the occurrence of bypass occlusion or confirmation of patency. All participants had undergone standard postoperative surveillance, including Doppler ultrasound or angiographic evaluation consistent with local guidelines. For patients with occlusion, repeated imaging confirmed graft failure.

Patients were classified into two groups based on bypass status at the time of enrollment. The case group included 33 patients with documented bypass graft occlusion, whereas the control group consisted of 14 patients with patent bypasses at their most recent imaging. Eighteen of the 47 patients (38%) had experienced an occlusion of a previous bypass graft; of these, eight were enrolled with a patent index graft at study inclusion and 10 with an occluded index graft. For all analyses, the index graft was defined as the bypass under evaluation at study inclusion, and time zero was the date of its implantation; prior occlusions refer to earlier grafts.

The study protocol was approved by the Ethics Committee of the Ludwig-Maximilians-University of Munich (approval number 20-206) and conducted in accordance with International Conference of Harmonization - Good Clinical Practice (ICH-GCP) guidelines and the Declaration of Helsinki.

### Targeted gene panel sequencing and CHIP detection

PB was drawn from each patient at the time of enrollment and collected in ethylenediaminetetraacetic acid tubes. Genomic DNA was extracted using the Maxwell 16 CSC Blood DNA Purification Kit (Promega; AS1321) or the Maxwell 16 RSC Cell DNA Purification Kit (Promega; AS1370) according to the manufacturer's instructions. CHIP-associated mutations were assessed using the AmpliSeq for Illumina Myeloid Sequencing Panel (Illumina), which covers hotspot regions and full coding sequences of 40 genes recurrently mutated in myeloid malignancies (complete gene list in Supplementary Table II, online only). This panel was selected because it includes all major CHIP-associated driver genes (*DNMT3A*, *TET2*, *ASXL1*, *JAK2*, *TP53*, *SF3B1*, and others) while also capturing less common but clinically relevant mutations.

Libraries were prepared according to the manufacturer's protocol and paired-end sequenced on a MiSeq sequencer (Illumina) using the MiSeq Reagent Kit V2 (500 cycles). Next generation sequencing data analysis was performed using the Local Run Manager (version 1.0.0.7; Illumina) with the *Generate FASTQ* (version 2.0.01.17) and *DNA Amplicon* (version 2.1.0.19) applications. GRCh37 (hg19) served as the reference genome for alignment and variant calling. Variant calling was performed with Illumina's BaseSpace Variant Interpreter, based on Pisces 5.2.9.23. Samples with a mean amplicon coverage below 1000× were excluded to ensure reliable detection of low-frequency variants. We defined CHIP operationally as the presence of somatic variants at ≥1% variant allele frequency (VAF) in genes recurrently mutated in hematologic malignancies, in the absence of cytopenia or hematological neoplasms,^14^ consistent with established criteria. We note that the current World Health Organization 2022 classification recommends a VAF threshold of ≥2% for CHIP; however, our amplicon-based deep sequencing approach with >1000× coverage reliably detects variants down to 1% VAF, and we chose this lower threshold to maximize sensitivity in this exploratory pilot study. Variants that were synonymous, intronic, or documented as germline polymorphisms in gnomAD were excluded.

The known *ASXL1* c.1934dupG artifact, which can arise from polymerase chain reaction-based library preparation, was identified and excluded from analysis. Each detected mutation is reported with gene name, specific variant notation according to the Human Genome Variation Society, VAF, and predicted functional consequence in Supplementary Table III (online only).

### Cell sorting

Fresh PB samples (8 mL, sodium heparin) were diluted 1:10 with 5× RBC lysis buffer (BD Biosciences) and incubated for 20 minutes at room temperature. Cells were stained with fluorochrome-conjugated antibodies for 20 minutes at 4 °C (Supplementary Table IV, online only) and sorted using the MoFlo Astrios EQ cell sorter (Beckman Coulter) into CD14+ monocytes, CD15+ granulocytes, CD3+ T cells, CD19+ B cells, and CD56+ natural killer cells. Sorted populations achieved >95% purity. Genomic DNA from sorted fractions was extracted and sequenced as described above (Supplementary Table V, online only).

### Cytokine measurement

Plasma cytokine levels were determined from the same PB samples used for targeted sequencing. Interleukin (IL)-6 levels were measured using the electrochemiluminescence immunoassay Elecsys IL-6 on a Cobas 8000 e801 analyzer (Roche Diagnostics; detection range, 1.5-5000 pg/mL). Plasma levels of IL-1β, IL-10, and tumor necrosis factor α were assessed by enzyme-linked immunosorbent assay (BD Biosciences) on a Synergy HTX multimode reader (BioTek/Agilent; detection range, 7.8-500 pg/mL).

### Statistical analysis

Continuous variables are reported as median with interquartile range (IQR, 25th-75th percentiles). Group comparisons for continuous variables used the nonparametric Mann-Whitney *U* test. Categorical variables are presented as absolute and relative frequencies and compared using the Fisher exact test.

To investigate associations of CHIP status and *TET2* mutations with bypass occlusion, time-to-event analyses were performed. Time zero was defined as the date of bypass surgery, and the event was bypass graft occlusion. Patients with patent grafts were censored at last follow-up. Because CHIP status was ascertained at enrollment rather than at the time of surgery, the Kaplan-Meier and Cox analyses rely on the assumption that clonal hematopoiesis was present before bypass implantation. This assumption is biologically plausible given the typically slow expansion kinetics of CHIP clones, which persist and expand over years to decades,^15^ but cannot be directly verified in our design.

Differences in time to bypass occlusion according to CHIP status or *TET2* mutation were assessed using Kaplan-Meier curves with log-rank test statistics. Associations were further examined by separate multivariable Cox proportional hazards regression models for each exposure. Given the small sample size (n = 47, 33 events) and the resulting constraint on events per variable, we limited covariate adjustment to diabetes and smoking status (never, former, and current) in the primary models to avoid overfitting. Besides smoking and diabetes, coronary heart disease (CHD) was included in sensitivity analyses but not as a covariate in the primary analysis because it represents a likely mediator on the hypothesized pathway from *TET2* to cardiovascular outcomes rather than a confounder (see the Discussion section). To improve model stability regarding the sample size, an additional sensitivity analysis was conducted by performing a Cox regression with Firth's penalized likelihood. The proportional hazards assumption was assessed by visual inspection of log(–log[survival]) plots.

To account for multiple testing of two pre-specified primary exposures (overall CHIP status and *TET2* mutation), *P* values from regression analyses less than the Bonferroni-corrected threshold α = 0.025 were considered statistically significant. We note that this correction is conservative because the two hypotheses are nested and correlated (*TET2* is a subset of CHIP), but we applied it to strengthen inferential rigor. All statistical analyses were performed using R version 4.5.1 (R Foundation for Statistical Computing) and GraphPad Prism 9 (Dotmatics).

## Results

### Patient cohort

The study included 47 predominantly male (81%) patients with a median age of 69 years (IQR: 65-77 years) who had undergone bypass grafting for PAD (Table). Of 45 patients with available bypass graft data, 37 (82%) received femoropopliteal and 8 (18%) femorocrural bypass (Supplementary Table VI, online only). Neither conduit type nor bypass level differed significantly between the occlusion and patency groups (prosthetic conduit: 74% vs 64%, *P* = .50; femorocrural level: 23% vs 7%, *P* = .40). Thirty-three patients (70.2%) experienced bypass graft occlusion after a median time of 26 months from surgery (IQR: 9-61 months). The median follow-up was 29 months (IQR: 12-48 months), with no significant difference between groups (*P* = .650). Because peripheral blood was sampled at presentation with occlusion or at a follow-up visit confirming patency, this interval also reflects the time from surgery to CHIP assessment; 10 of 47 patients (21.3%) were sampled more than 5 years after bypass surgery.

**Table:** Patient characteristics for the total cohort and stratified by bypass status

Characteristics	Total sample (N = 47)	Open bypass graft (n = 14)	Occluded bypass graft (n = 33)	*P* value
Follow-up time, months	29 (12-48)	30 (19-36)	26 (8.8-61)	.650
Age, years	69 (65-77)	68 (65-71)	72 (64-77)	.300
CHIP				1.000
No	33 (0.702)	10 (0.714)	23 (0.697)	
Yes	14 (0.298)	4 (0.286)	10 (0.303)	
*TET2* mutation				.303
No	42 (0.894)	14 (1)	28 (0.848)	
Yes	5 (0.106)	0 (0)	5 (0.152)	
Sex				.419
Men	38 (0.809)	10 (0.714)	28 (0.848)	
Women	9 (0.191)	4 (0.286)	5 (0.152)	
Hypertension				.700
No	10 (0.213)	2 (0.143)	8 (0.242)	
Yes	37 (0.787)	12 (0.857)	25 (0.758)	
Diabetes				.516
No	29 (0.617)	10 (0.714)	19 (0.576)	
Yes	18 (0.383)	4 (0.286)	14 (0.424)	
CHD				.288
No	34 (0.723)	12 (0.857)	22 (0.667)	
Yes	13 (0.277)	2 (0.143)	11 (0.333)	
Stroke				1.000
No	42 (0.913)	13 (0.929)	29 (0.906)	
Yes	4 (0.087)	1 (0.071)	3 (0.094)	
Smoking				.605
Current	18 (0.383)	7 (0.500)	11 (0.333)	
Former	16 (0.340)	4 (0.286)	12 (0.364)	
Never	13 (0.277)	3 (0.214)	10 (0.303)	
PAD stage (Fontaine classification)				<.001
II	20 (0.426)	14 (1)	6 (0.182)	
III	21 (0.447)	0 (0)	21 (0.636)	
IV	6 (0.128)	0 (0)	6 (0.182)	

*CHD*, Coronary heart disease; *CHIP*, clonal hematopoiesis of indeterminate potential; *PAD*, peripheral arterial disease.

Continuous variables are presented as median with the corresponding interquartile range and compared using the Mann-Whitney *U* test. Categorical variables are presented as absolute and relative frequencies and evaluated using the Fisher exact test.

The only significant baseline difference between the two groups was PAD stage: all control patients were Fontaine stage II, whereas the occlusion group was predominantly stage III or IV (27 of 33), with the remaining six patients at stage II (*P* < .001). Laboratory values and concomitant medications are shown in Supplementary Tables VII and VIII (online only).

### Prevalence of CHIP in the study cohort

CHIP was detected in 14 of 47 patients (29.8%). Prevalence increased with age: 0% in patients aged <60 years (n = 5), 25.0% in the 60- to 69-year group, 37.5% in the 70- to 79-year group, and 50.0% in patients aged ≥80 years (Fig 1, *A*). These rates exceeded published prevalence estimates for the general population across all age groups.^10^^,^^16^^,^^17^ Among the 14 CHIP carriers, 11 harbored a single mutation, one had two variants, and two had four variants (Supplementary Table III, online only).

**Fig 1:**
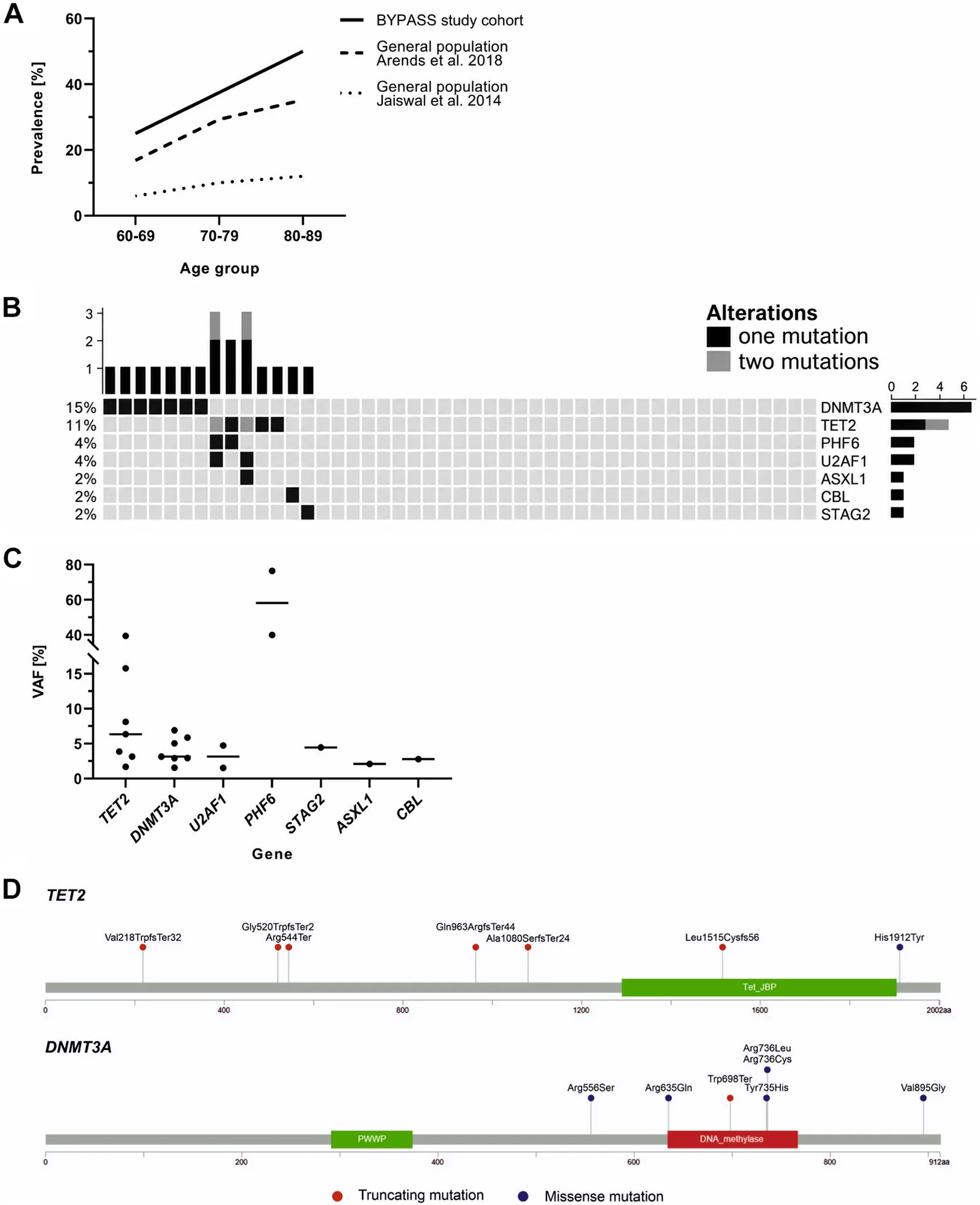
CHIP mutations in the study cohort. **A,** Prevalence of CHIP-associated mutations in patients of the cohort (n = 47) compared with previously published prevalence rates within the general population. **B,** Distribution of genetic alterations in the patient cohort. The oncoprint shows only those genes of the 40-gene AmpliSeq for Illumina Myeloid Sequencing Panel in which mutations were detected. **C,** Distribution of variant allele frequencies (VAFs) of all CHIP-related variants detected in the study cohort. **D,** Lollipop plot depicting type and location of mutations found in the most frequently mutated genes, TET2 and DNMT3A. *CHIP*, Clonal hematopoiesis of indeterminate potential.

Mutations in *TET2* (seven mutations in five patients, 10.6%) and *DNMT3A* (seven mutations in seven patients, 14.9%) were most frequent (Fig 1, *B* and *C*). *TET2* and *DNMT3A* mutations did not co-occur in any individual. Median VAFs were 6.4% (range, 1.7%-39.5%) for *TET2* and 3.1% (range, 1.6%-6.9%) for *DNMT3A*. The highest VAFs were observed for *PHF6* mutations (40.0% and 76.4%). Notably, six of seven *TET2* mutations (85.7%) were truncating (frameshift/nonsense), consistent with loss of function, while *DNMT3A* mutations were predominantly missense (6 of 7; 85.7%) (Fig 1, *D*).

### CHIP mutations in different cell lineages

To characterize the distribution of CHIP mutations across hematopoietic lineages, PB leukocytes from five CHIP carriers with bypass occlusion were sorted by fluorescence-activated cell sorting and individually sequenced (Supplementary Table V, online only). CHIP-related variants were predominantly present in monocytes (median VAF, 9.5%), granulocytes (8.0%), natural killer cells (4.5%), and B cells (2.0%). In contrast, CHIP variants were largely absent from T cells, with a significantly lower mean VAF compared with all other cell types (*P* < .05; Fig 2, *A*). Analysis of *TET2* mutations specifically showed a similar distribution, with no *TET2* mutations detectable in T cells and only three of seven variants found in B cells (Fig 2, *B*). This myeloid-predominant distribution is consistent with the known lineage tropism of *TET2*-mutant clones.

**Fig 2:**
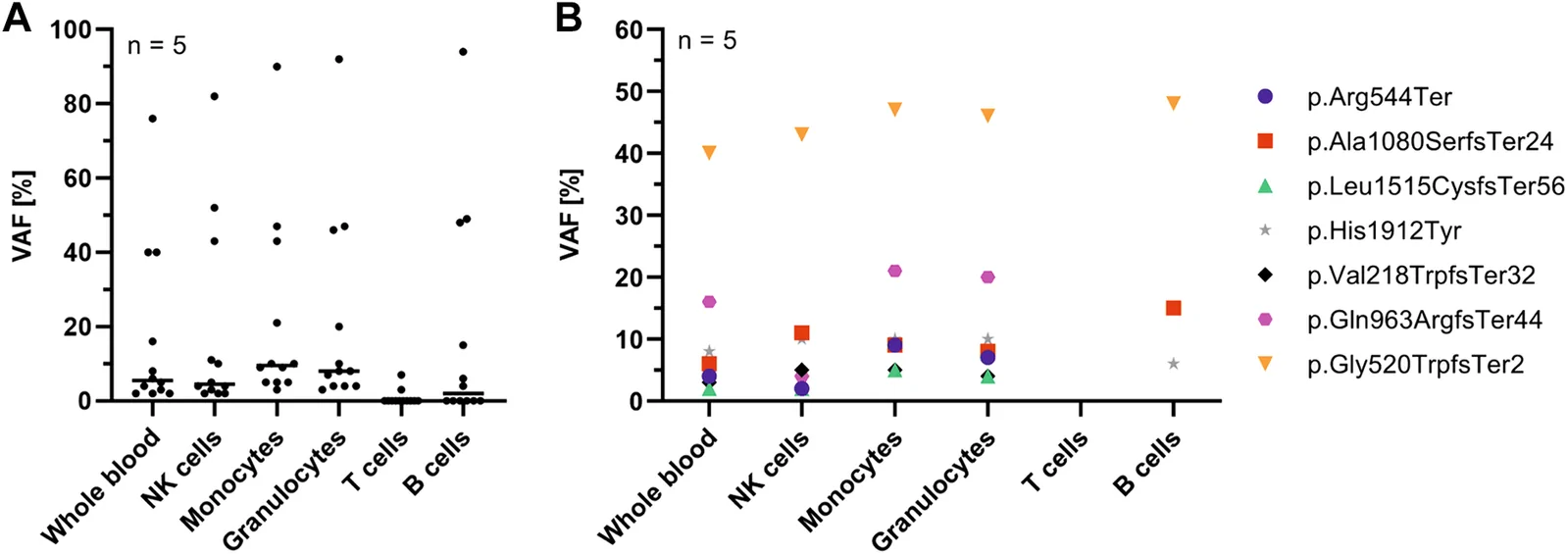
CHIP-related mutations in hematopoietic subpopulations. CHIP-related variants in hematopoietic subpopulations, obtained by flow-sorting of peripheral blood from five patients with a bypass graft occlusion, were analyzed individually. **A,** Median VAF of CHIP-associated mutations detected in the different cell lineages. **B,** VAF of TET2 CHIP variants in hematopoietic subpopulations. *CHIP*, Clonal hematopoiesis of indeterminate potential; *NK*, natural killer; *VAF*, variant allele frequency.

### Associations between CHIP, *TET2*, and bypass graft occlusion

Overall CHIP prevalence was similar between groups (30.3% in the occlusion group vs 28.6% in the open bypass group; *P* = 1.0; Fig 3, *A*). The prevalence of CHIP mutations in patients aged between 60 and 69 years was 25.0% in both groups. In the 70- to 79-year age group, patients with a bypass graft occlusion showed a higher prevalence (41.7%) than those with an open bypass graft (25%). Forty percent of patients older than 80 years with a bypass graft occlusion had CHIP. However, because of the small number of subjects with an open bypass in this age group, a comparison of the two groups was not feasible. In patients younger than 60 years, no CHIP-associated variants were identified at all (Fig 3, *B*). Median VAFs trended higher in the occlusion group (4.7% vs 3.0%), but this difference did not reach significance (Fig 3, *C*). In patients with patent bypasses, all four CHIP mutations were in *DNMT3A* (4 of 4, 100%), whereas in the occlusion group, *TET2* was the most frequently mutated gene (7 of 17 mutations, 41%), followed by *DNMT3A* (3 of 17, 18%) (Fig 3, *D*). All five *TET2*-mutant patients belonged to the occlusion group; their individual clinical, bypass-graft, and outcome characteristics are summarized in Supplementary Table IX (online only).

**Fig 3:**
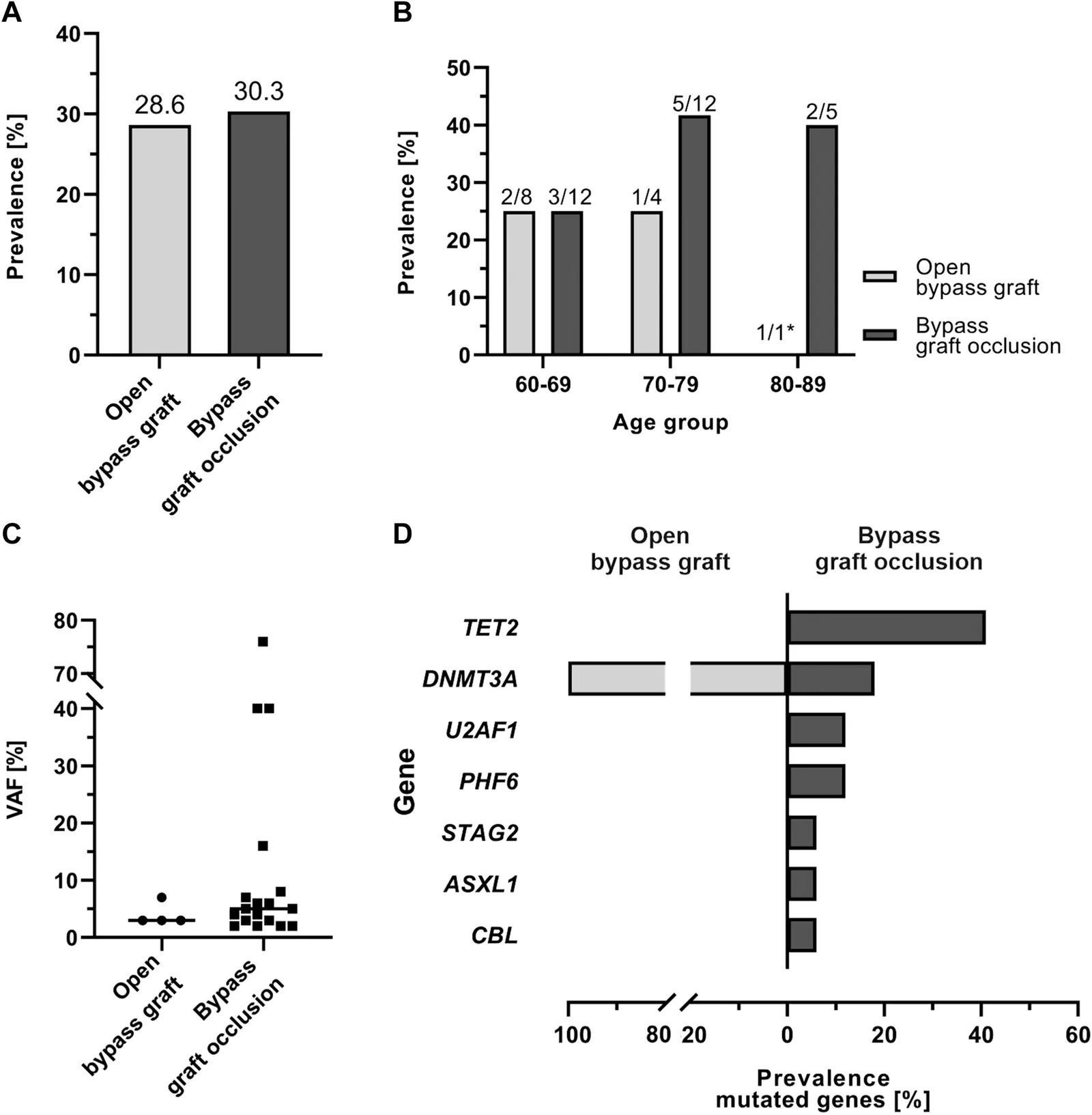
Comparison of CHIP mutations in patients with bypass graft occlusion and open bypass graft. **A,** Comparison of CHIP prevalence between the open bypass graft group (n = 14) and the bypass graft occlusion group (n = 33). **B,** Prevalence of CHIP-associated mutations, according to age, in patients with an open bypass graft or with bypass graft occlusion. The numbers above the *bars* show the number of CHIP carriers per total analyzed patients in this age group. Data for the age group <60 years (n = 5) are not shown because no mutations were detected. ∗Only one proband with an open bypass graft was recruited for this age group. As this value is not representative, it was excluded from the bar chart. **C,** Comparison of variant allele frequencies (*VAF*s) of CHIP-associated mutations detected in the open bypass graft group and the bypass graft occlusion group. **D,** Prevalence of CHIP-related variants per affected gene identified in patients of the open bypass graft group and the bypass graft occlusion cohort. In total, four mutations were found in the open bypass graft group and 17 mutations in patients with a bypass graft occlusion. *CHIP*, Clonal hematopoiesis of indeterminate potential.

CHIP-positive patients had shorter time to graft failure compared with CHIP-negative patients (log-rank *P* = .042; Fig 4, *A*). Kaplan-Meier analysis (n = 44 patients with available follow-up data) revealed shorter time to bypass occlusion in patients with *TET2* mutations compared with those without (log-rank *P* = .003; Fig 4, *B*).

**Fig 4:**
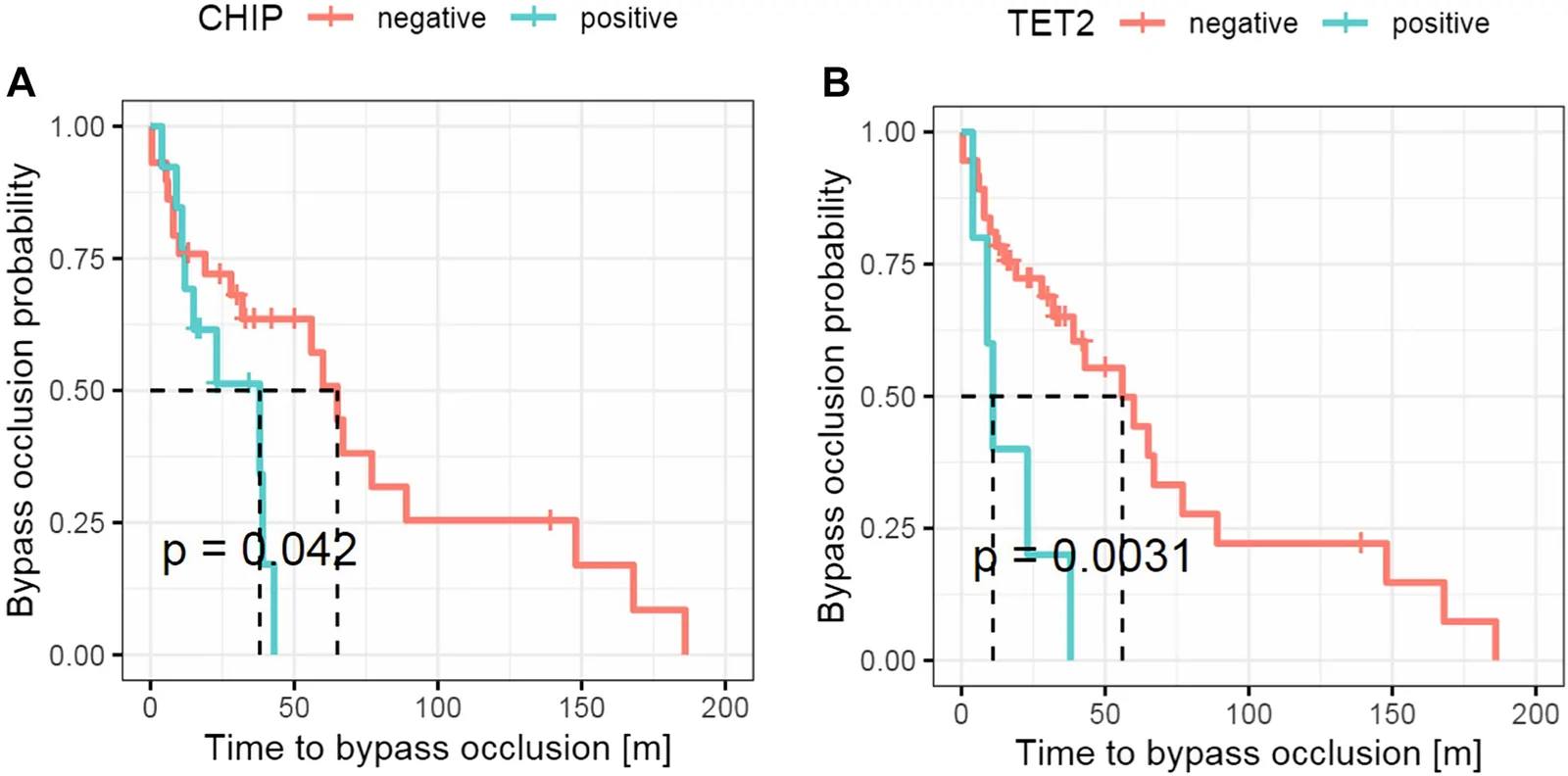
Impact of CHIP-related mutations on the patency rate of bypass grafts. Kaplan-Meier curves defined the time between the date of surgery and the date of bypass graft occlusion for n = 44 patients with available follow-up data. Patients with open bypass graft were censored at last follow-up. Statistical significance was assessed using the log-rank test. **A,** Analysis of patients stratified by the presence or absence of CHIP. **B,** Analysis of patients stratified by *TET2* mutation. *CHIP*, Clonal hematopoiesis of indeterminate potential.

In the primary multivariable Cox regression model adjusted for diabetes and smoking status, *TET2* mutation was significantly associated with bypass occlusion (hazard ratio [HR], 6.70; 95% confidence interval [CI], 1.96-22.96; *P* = .003; Fig 5). Overall CHIP status showed a positive but nonsignificant association after Bonferroni correction (HR, 2.79; 95% CI, 0.96-8.11; *P* = .059).

**Fig 5:**
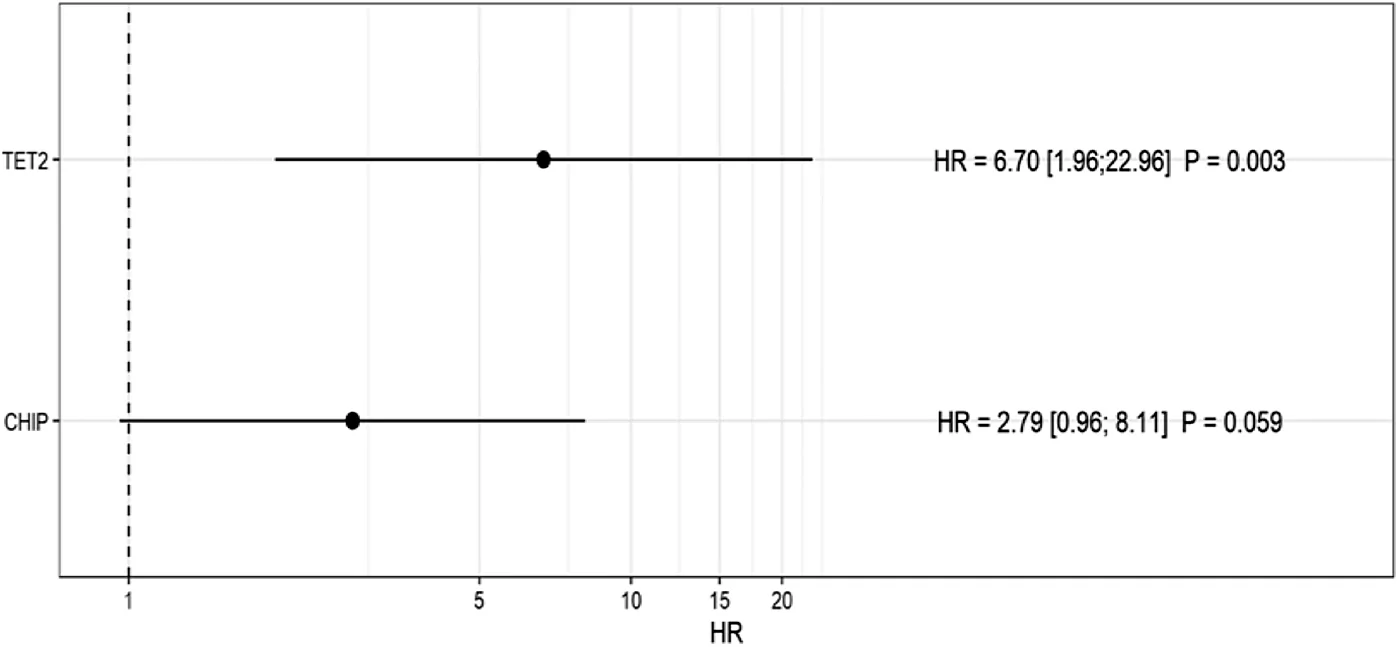
Multivariable Cox proportional hazards regression model for the associations between TET2 and CHIP and bypass occlusion. Hazard ratios (*HRs*) with 95% confidence intervals (CIs), adjusted for diabetes and smoking status. Estimates are presented on a log scale. Statistical significance was defined at a Bonferroni-corrected threshold of *P* < .025. *CHIP*, Clonal hematopoiesis of indeterminate potential.

In additional sensitivity analyses (including CHD) and using Firth's penalized likelihood, the *TET2* association remained robust (Supplementary Table X, online only). CHD prevalence was numerically higher among *TET2*-positive patients (3 of 5, 60%) compared with *TET2*-negative patients (10 of 42, 24%), consistent with the known *TET2*-CHD mechanistic link.

Given the small number of *TET2*-positive patients (n = 5), the point estimate of HR 6.70 should be interpreted with caution, as small-sample Cox regression is known to produce inflated hazard ratios. The wide CI (1.96-22.96) reflects this uncertainty.

### Cytokine profiles

Median IL-6 was highest among CHIP carriers with occluded bypass (median, 5.9 pg/mL vs 2.1 pg/mL in non-CHIP patients; Fig 6). IL-1β, IL-10, and tumor necrosis factor α levels were consistently low and within the normal range (data not shown). Although these differences did not reach statistical significance given the sample size, the numerical elevation of IL-6 in CHIP carriers with graft occlusion is consistent with the hypothesis that *TET2* deficiency contributes to proinflammatory pathways relevant to bypass failure.

**Fig 6:**
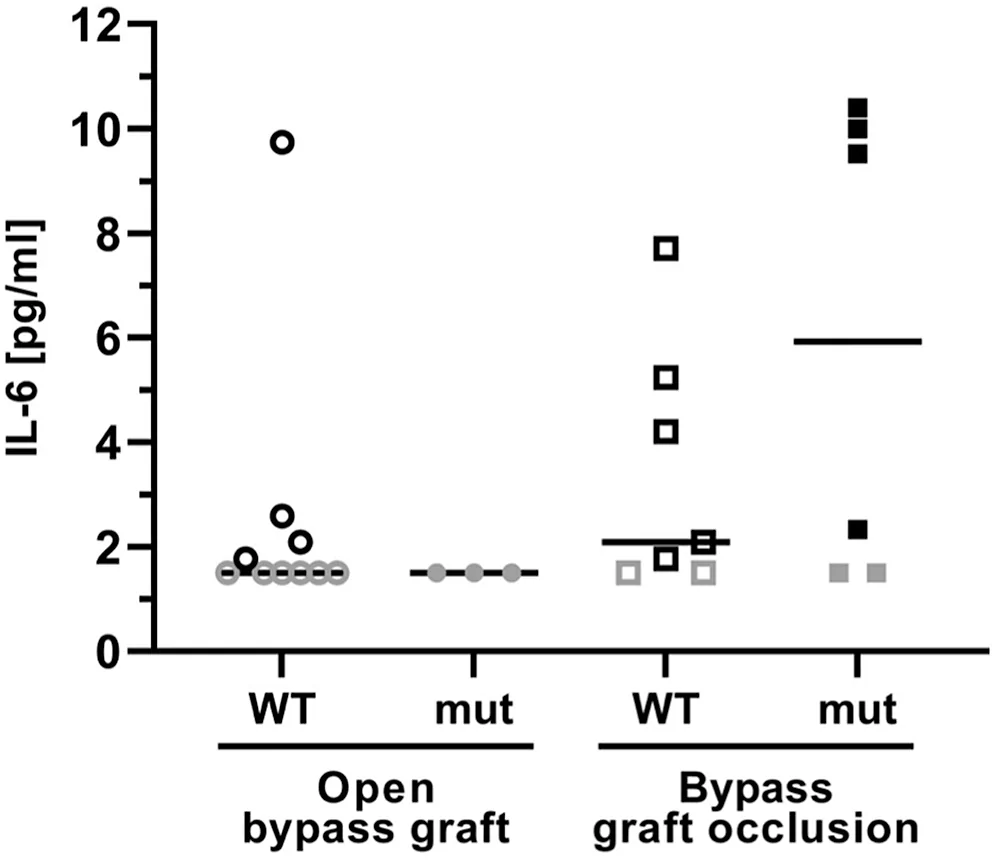
Cytokine profile. Plasma IL-6 concentrations (pg/mL) at the time of enrollment are shown for patients stratified by bypass occlusion and CHIP status. Each symbol represents an individual patient, with the *horizontal lines* in the boxplots indicating the median IL-6 level in each subgroup. *CHIP*, Clonal hematopoiesis of indeterminate potential; *IL*, interleukin; *WT*, wild-type.

## Discussion

In this multicenter, retrospective cohort pilot study, we provide the first evidence of an association between CHIP, particularly *TET2* mutations, and peripheral bypass graft occlusion. Although the association between CHIP and atherosclerotic cardiovascular disease has been established through large epidemiological studies,^10^^,^^11^ mechanistic mouse models,^18^ and the therapeutic proof of concept provided by the CANTOS trial,^19^ its impact on the outcomes of peripheral vascular surgical interventions has not previously been investigated. Our finding that *TET2* mutations are associated with bypass graft occlusion (HR, 6.70; *P* = .003) therefore extends the cardiovascular phenotype of CHIP beyond native atherosclerosis and addresses an important gap in the prediction of surgical outcomes in patients with PAD. The available literature most closely related to our findings falls into three categories.

First, CHIP mutations have been detected in atherosclerotic plaque tissue of patients with PAD, demonstrating that mutant hematopoietic clones infiltrate peripheral arterial lesions, but surgical outcomes were not examined.^20^ Second, recent studies have linked CHIP to adverse outcomes after coronary bypass surgery: a two-fold increased risk for major adverse cardiac events was observed in CHIP carriers after cardiac surgery,^21^ and CHIP-associated cardiovascular mortality was demonstrated after coronary artery bypass graft in a separate cohort (n = 497).^22^

Third, a causal, unidirectional association between CHIP and de novo femoral atherosclerosis was established using Mendelian randomization in the Progression of Early Subclinical Atherosclerosis (PESA) study cohort (n = 3692).^23^ Our study extends this body of evidence by showing that CHIP may also affect the patency of surgical bypass grafts in the peripheral vascular bed—a pathological process distinct from native vessel atherosclerosis, as graft occlusion involves intimal hyperplasia and inflammatory remodeling of non-native conduits.

CHIP prevalence in our cohort (29.8%) exceeded published estimates for the general population across all age groups,^10^^,^^16^^,^^17^ consistent with accumulating evidence that patients with PAD harbor CHIP at elevated rates.^20^^,^^24^ However, differences in sequencing sensitivity must be considered: our amplicon-based method with >1000× coverage detects variants down to 1% VAF, whereas studies using whole-exome sequencing with lower read depth may underestimate CHIP prevalence.^10^^,^^25^ A notable finding of our study was the gene-specific distribution of CHIP mutations between the two patient groups. Although *DNMT3A* was the only mutated gene in patients with patent bypasses, *TET2* dominated in the occlusion group (41% of all mutations), and all five *TET2*-mutant patients had graft occlusion.

The predominance of *TET2* mutations among patients with bypass graft occlusion is biologically plausible. Loss of *TET2* function promotes an inflammatory phenotype through NLRP3 inflammasome activation, resulting in increased IL-1β and IL-6 secretion by macrophages.^18^^,^^26^ This inflammatory cascade may accelerate both atherosclerosis in the run-off vessels and intimal hyperplasia at the graft-vessel anastomosis, the two principal mechanisms of late graft failure. These observations also have potential therapeutic implications. In a post hoc analysis of the CANTOS trial, patients with *TET2*-driven CHIP derived disproportionate benefit from IL-1β inhibition with canakinumab (HR, 0.38 for major adverse cardiac events), suggesting that *TET2*-associated inflammation may be therapeutically targetable.^19^ Moreover, colchicine prevented accelerated atherosclerosis in a preclinical model of *TET2*-mutant clonal hematopoiesis, further supporting the rationale for anti-inflammatory interventions.^27^

The myeloid-predominant distribution of CHIP mutations we observed—with high VAFs in monocytes and granulocytes but near-absence in T cells—aligns with the known lineage tropism of *TET2*-mutant clones and is consistent with a macrophage-mediated inflammatory mechanism.^18^^,^^26^^,^^28^^,^^29^ Our finding of numerically elevated IL-6 levels in CHIP carriers with occluded grafts further supports this inflammatory hypothesis, although the sample size precluded formal statistical testing.

We excluded CHD from the primary Cox regression model because published mechanistic evidence suggests that it lies on the pathway between *TET2*-associated inflammation and cardiovascular outcomes, making it a likely mediator rather than a confounder. We note that this reasoning rests on a hypothesized pathway that our data cannot verify. Accordingly, the primary model was adjusted only for diabetes and smoking status, the strongest confounders of both CHIP prevalence and graft outcomes. In a sensitivity analysis additionally adjusting for CHD, the association between *TET2* mutations and bypass graft occlusion was modestly attenuated but remained statistically significant, consistent with partial mediation rather than confounding.

PAD severity differed markedly between groups: control patients were exclusively Fontaine stage II, whereas the occlusion group was predominantly stage III or IV. This distribution largely reflects the clinical consequence of graft occlusion, which itself precipitates chronic limb-threatening ischemia, rather than a pre-existing difference between groups; a patent graft implies, conversely, that the limb did not progress to critical ischemia. Fontaine stage at enrollment is therefore better regarded as a downstream correlate of the outcome than as a baseline confounder. Recognizing this, we nonetheless assessed its influence in a sensitivity analysis; after adjustment for PAD stage, the association between *TET2* mutation and bypass occlusion persisted (HR, 6.13; 95% CI, 1.71-21.67; *P* = .005; Supplementary Table X, online only). Fontaine stage at the index bypass, which would provide a cleaner measure of baseline severity, was not retrievable in this retrospective cohort, so this analysis cannot fully exclude an effect of baseline severity.

Whether *TET2* mutations independently promote graft occlusion or partly act through accelerated PAD progression requires prospective studies with PAD staging at the time of bypass surgery. These findings suggest a clinically relevant pattern: the time between graft implantation and occlusion was substantially shorter in patients with *TET2* mutations, raising the possibility that CHIP-associated inflammation contributes to earlier graft failure. Occlusions among CHIP carriers occurred in both prosthetic and autologous conduits, consistent with a systemic inflammatory mechanism rather than one confined to the native vessel wall; all five *TET2*-mutant patients, however, had received prosthetic (or undetermined) conduits, so whether this extends to vein-graft failure remains open.

If confirmed in larger prospective cohorts, *TET2* mutation status may represent a clinically useful biomarker for risk stratification before bypass surgery and identify patients who could benefit from intensified surveillance or targeted anti-inflammatory therapy.

In this regard, preclinical data showing that colchicine prevents accelerated atherosclerosis in *TET2*-mutant clonal hematopoiesis^27^ and the clinical evidence that *TET2*-CHIP patients derive disproportionate benefit from IL-1β inhibition^19^ provide a rational basis for exploring such interventions in patients with PAD with bypass grafts.

### Limitations

This study has several limitations, and the findings should be considered preliminary and hypothesis-generating. First, the small sample size (n = 47), including only five patients with *TET2* mutations, limits statistical power and may have inflated the estimated effect size. Although the observed association between *TET2* mutations and bypass graft occlusion was strong (HR, 6.70), the wide CI (1.96-22.96) indicates substantial uncertainty, and larger studies are required to obtain more precise estimates. The limited sample size also precluded meaningful subgroup analyses and limits the generalizability of our findings. Second, CHIP was assessed at a single time point after bypass surgery using a targeted 40-gene myeloid panel, which may miss approximately 10% of CHIP-positive individuals carrying mutations in genes outside the panel. Our VAF threshold of ≥1% was lower than the World Health Organization 2022 recommendation of ≥2%, maximizing sensitivity at the cost of potentially including very small clones of uncertain biological significance. Furthermore, because CHIP was assessed after bypass surgery, the interval between surgery and blood sampling varied across patients, introducing the possibility of temporal misclassification. However, longitudinal studies have consistently shown that CHIP clones, particularly *TET2*-mutant clones, arise and expand gradually at rates of a few percent per year, such that a detectable clone typically reflects a decade or more of growth;^15^^,^^30^ de novo acquisition during the observed sampling interval (median, 29 months) is therefore unlikely.

Notably, all five *TET2*-mutant patients were assessed within 38 months of bypass surgery (Supplementary Table IX, online only). Nevertheless, CHIP status at the time of bypass surgery cannot be confirmed within the retrospective design of this study. Prospective studies with CHIP assessment at the time of bypass surgery are therefore required to eliminate this temporal ambiguity and validate the observed association.

Third, Fontaine stage at the index bypass, formal runoff scores, Wound, Ischemia, foot Infection (WIfI) classification, above- vs below-knee target designation, prior ipsilateral interventions, and quantitative measures of target-vessel and graft diameter were not systematically available for all 47 patients. Adjustment for these determinants of graft patency was therefore not possible, and residual confounding by baseline disease severity cannot be excluded.

No *TET2* mutations were identified among the eight patients with occluded autologous vein grafts. Given the superior baseline patency of vein conduits, a *TET2* signal in this subgroup would have been of particular interest; with only eight vein-graft failures, however, we could not determine whether this absence reflects a conduit-specific effect or the limited number of events. Larger cohorts enriched for autologous vein grafts—and capturing restenosis in addition to complete occlusion, given the shared inflammatory basis of intimal hyperplasia—will be required to address this question.

Finally, the retrospective design introduces the potential for survivor bias and informative censoring. In addition, a proportion of patients in both groups had experienced occlusion of an earlier bypass graft; because this includes patients enrolled with a patent index graft, the control group may be enriched for a predisposition to graft failure, which would bias the observed association toward the null. Based on our observed effect sizes, a prospective validation study including approximately 150 to 250 patients, with CHIP assessment at the time of bypass surgery and enrichment for autologous vein grafts, would likely provide adequate power to confirm these findings.

## Conclusions

In conclusion, this pilot study provides preliminary evidence that clonal hematopoiesis, and in particular *TET2* mutations, may constitute a novel risk factor for early peripheral bypass graft occlusion, potentially reflecting inflammatory processes. If validated, *TET2* mutation status could serve as an additional biomarker for the individual risk of graft failure. Our findings suggest that the association of CHIP with vascular outcomes may extend beyond long-term modulation of native vessel atherosclerosis to include more short-term effects on surgical graft patency. Prospective validation in larger multicenter cohorts, ideally with CHIP assessment at the time of bypass surgery, is the essential next step to confirm these findings and evaluate whether targeted anti-inflammatory interventions can improve graft outcomes in CHIP-positive patients with PAD.

## Author Contributions

Conception and design: ES, RC

Analysis and interpretation: ES, MH, DF, SD, EK, BM, MT, AH, RC

Data collection: ES, DD, DJ

Writing the article: ES, MH, DF, RC

Critical revision of the article: ES, MH, DF, SD, DD, DJ, EK, BM, MT, AH, RC

Final approval of the article: ES, MH, DF, SD, DD, DJ, EK, BM, MT, AH, RC

Statistical analysis: ES, DF, RC

Obtained funding: ES, RC

Overall responsibility: RC

## Funding

This study was financially supported by intramural research grants (“Interdisciplinary study of clonal hematopoiesis of indeterminate potential (CHIP) in patients with femoropopliteal or femorocrural bypass occlusions”) from the Medical Faculty of the University of Augsburg.

## Disclosures

None.
