# Supplementary material for: TET2-mutant clonal hematopoiesis is associated with peripheral bypass graft occlusion: A multicenter retrospective cohort pilot study
Source: JVS Vasc Sci. 2026 Aug 23;7:100442. doi: 10.1016/j.jvssci.2026.100442 (PMC13634526; doi:10.1016/j.jvssci.2026.100442)
Supplement: Supplementary file 1 [file mmc1.docx]

***TET2*-mutant clonal hematopoiesis is associated with peripheral bypass graft occlusion: a multicenter retrospective cohort pilot study**

Elena Streck^1^, Manuela Harloff^2^, Dennis Freuer^3^, Sebastian Dintner^2^, Dimitrios Dedegkikas^4^, Dominik Jost^5^, Elisabeth Kling^6^, Bruno Märkl^2^, Martin Trepel^7^, Alexander Hyhlik-Dürr^1^, Rainer Claus^2,7,8,§^

^1^ Vascular Surgery, Faculty of Medicine, University of Augsburg, Stenglinstrasse 2, 86156 Augsburg, Germany

^2^ Pathology, Faculty of Medicine, University of Augsburg, Stenglinstrasse 2, 86156 Augsburg, Germany

^3^ Epidemiology, Faculty of Medicine, University of Augsburg, Stenglinstrasse 2, 86156 Augsburg, Germany

^4^ Klinik Günzburg, Lindenallee 2, 89312 Günzburg, Germany

^5^ St. Elisabethen-Klinikum, Elisabethenstrasse 15, 88212 Ravensburg, Germany

^6^ Microbiology, Faculty of Medicine, University of Augsburg, Stenglinstrasse 2, 86156 Augsburg, Germany

^7^ Hematology and Oncology, Faculty of Medicine, University of Augsburg, Stenglinstrasse 2, 86156 Augsburg, Germany

^8^ Comprehensive Cancer Center Augsburg, Faculty of Medicine, University of Augsburg, Stenglinstrasse 2, 86156 Augsburg, Germany

**Supplemental Table S1.** Inclusion and exclusion criteria of study

| **Inclusion Criteria** | **Exclusion Criteria** |
| --- | --- |
| - ability to give informed consent to participate in the study - male or female subjects > 18years old with PAD and bypass graft - regular antithrombotic therapy: platelet aggregation inhibitors or anticoagulants - detection of the bypass graft patency or occlusion using computer tomography, angiography or ultrasound | - bedridden patients - presence of tumor, autoimmune disease or infections - immunosuppressive drugs - bypass occlusion for technical reasons or infections - no antiplatelet therapy or anticoagulants |

**Supplemental Table S2.** List of genes, analyzed with the AmpliSeq for Illumina

Myeloid Panel

| **Gene** | **Target region** | **Gene** | **Target region** | **Gene** | **Target region** | **Gene** | **Target region** |
| --- | --- | --- | --- | --- | --- | --- | --- |
| ABL1 | HS | DNMT3A | HS | MPL | HS | SETBP1 | HS |
| ASXL1 | FG | FLT3 | HS | MYD88 | HS | SF3B1 | HS |
| BCOR | FG | GATA2 | HS | NF1 | FG | SH2B3 | FG |
| BRAF | HS | HRAS | HS | NPM1 | HS | SRSF2 | HS |
| CALR | FG | IKZF1 | FG | NRAS | HS | STAG2 | FG |
| CEBPA | FG | IDH1 | HS | PHF6 | FG | TET2 | FG |
| CBL | HS | IDH2 | HS | PRPF8 | FG | TP53 | FG |
| CSF3R | HS | JAK2 | HS | PTPN11 | HS | U2AF1 | HS |
| ETV6 | FG | KIT | HS | RB1 | FG | WT1 | HS |
| EZH2 | FG | KRAS | HS | RUNX1 | FG | ZRSR2 | FG |

FG: Full gene; HS: Hotspot regions

**Supplemental Table S3.** Detailed annotations of detected CHIP variants

| **No.** | **Patient-ID** | **Bypass graft closure** | **Gene** | **Exon** | **HGVS.c** | **HGVs.p** | **Annotation** | **Alt Allele  Depth** | **Total Read  Depth** | **VAF %** | **Biological effect*** |
| --- | --- | --- | --- | --- | --- | --- | --- | --- | --- | --- | --- |
| 1 | BRC20/0001 | + | TET2 | 3 | c.1557dup | p.Gly520TrpfsTer2 | frameshift_insertion | 873 | 2212 | 40 | likely loss-of-function |
|  |  |  | PHF6 | 10 | c.1030_1031del | p.Ser344Ter | stop_gained | 1080 | 1413 | 76 | not in database** |
| 2 | BRC20/0002 | + | ./. | | | | | | | | |
| 3 | BRC20/0004 | + | ./. | | | | | | | | |
| 4 | BRC20/0005 | + | ./. | | | | | | | | |
| 5 | BRC21/0001 | + | ./. | | | | | | | | |
| 6 | BRC21/0002 | + | ./. | | | | | | | | |
| 7 | BRC21/0003 | + | ./. | | | | | | | | |
| 8 | BRC21/0004 | + | ./. | | | | | | | | |
| 9 | BRC21/0005 | + | STAG2 | 12 | c.1108C>T | p.Arg370Trp | missense_variant | 14 | 314 | 4 | unknown |
| 10 | BRC21/0007 | + | TET2 | 3 | c.1630C>T | p.Arg544Ter | stop_gained | 98 | 2532 | 4 | likely loss-of-function |
|  |  |  | TET2 | 3 | c.3236dup | p.Ala1080SerfsTer24 | frameshift_insertion | 98 | 1544 | 6 | likely loss-of-function |
|  |  |  | U2AF1 | 6 | c.470A>G | p.Gln157Arg | missense_variant | 144 | 3041 | 5 | likely gain-of-function |
|  |  |  | PHF6 | 2 | c.92dup | p.Leu31PhefsTer5 | frameshift_insertion | 504 | 1261 | 40 | not in database** |
| 11 | BRC21/0008 | + | ./. | | | | | | | | |
| 12 | BRC21/0009 | + | ./. | | | | | | | | |
| 13 | BRC21/0010 | + | TET2 | 3 | c.2888del | p.Gln963ArgfsTer44 | frameshift_deletion | 500 | 3169 | 16 | likely loss-of-function |
| 14 | BRC21/0011 | + | TET2 | 3 | c.651del | p.Val218TrpfsTer32 | frameshift_deletion | 75 | 2399 | 3 | likely loss-of-function |
| 15 | BRC21/0012 | + | TET2 | 11 | c.4544del | p.Leu1515CysfsTer56 | frameshift_deletion | 46 | 2727 | 2 | likely loss-of-function |
|  |  |  | TET2 | 11 | c.5734C>T | p.His1912Tyr | missense_variant | 244 | 3007 | 8 | unknown |
|  |  |  | U2AF1 | 6 | c.470A>G | p.Gln157Arg | missense_variant | 55 | 3614 | 2 | likely gain-of-function |
|  |  |  | ASXL1 | 12 | c.1249C>T | p.Arg417Ter | stop_gained | 49 | 2335 | 2 | likely loss-of-function |
| 16 | BRC21/0013 | + | ./. | | | | | | | | |
| 17 | BRC21/0014 | + | ./. | | | | | | | | |
| 18 | BRC21/0015 | + | ./. | | | | | | | | |
| 19 | BRC21/0016 | + | ./. | | | | | | | | |
| 20 | BRC21/0017 | + | ./. | | | | | | | | |
| 21 | BRC21/0018 | + | ./. | | | | | | | | |
| 22 | BRC21/0020 | + | CBL | 8 | c.1211G>A | p.Cys404Tyr | missense_variant | 36 | 1295 | 3 | unknown |
| 23 | BRC21/0021 | + | ./. | | | | | | | | |
| 24 | BRC21/0022 | + | DNMT3A | 16 | c.1904G>A | p.Arg635Gln | missense_variant | 197 | 3911 | 5 | likely loss-of-function |
| 25 | BRC21/0023 | + | ./. | | | | | | | | |
| 26 | BRC21/0024 | + | ./. | | | | | | | | |
| 27 | BRC21/0025 | - | DNMT3A | 15 | c.1668G>T | p.Arg556Ser | missense_variant | 66 | 2099 | 3 | unknown |
| 28 | BRC21/0027 | + | ./. | | | | | | | | |
| 29 | BRC21/0028 | - | ./. | | | | | | | | |
| 30 | BRC21/0029 | + | ./. | | | | | | | | |
| 31 | BRC21/0031 | + | ./. | | | | | | | | |
| 32 | BRC21/0032 | + | DNMT3A | 18 | c.2094G>A | p.Trp698Ter | stop_gained | 147 | 2507 | 6 | likely loss-of-function |
| 33 | BRC21/0033 | - | DNMT3A | 19 | c.2207G>T | p.Arg736Leu | missense_variant | 48 | 1647 | 3 | likely loss-of-function |
| 34 | BRC21/0034 | - | ./. | | | | | | | | |
| 35 | BRC21/0035 | - | ./. | | | | | | | | |
| 36 | BRC21/0036 | + | ./. | | | | | | | | |
| 37 | BRC21/0037 | - | ./. | | | | | | | | |
| 38 | BRC21/0038 | + | DNMT3A | 19 | c.2203T>C | p.Tyr735His | missense_variant | 41 | 2647 | 2 | unknown |
| 39 | BRC21/0039 | - | ./. | | | | | | | | |
| 40 | BRC21/0040 | - | ./. | | | | | | | | |
| 41 | BRC22/0001 | + | ./. | | | | | | | | |
| 42 | BRC22/0002 | - | DNMT3A | 23 | c.2684T>G | p.Val895Gly | missense_variant | 212 | 3067 | 7 | not in database** |
| 43 | BRC22/0003 | - | ./. | | | | | | | | |
| 44 | BRC22/0004 | - | DNMT3A | 19 | c.2206C>T | p.Arg736Cys | missense_variant | 108 | 3671 | 3 | likely loss-of-function |
| 45 | BRC22/0005 | - | ./. | | | | | | | | |
| 46 | BRC22/0006 | - | ./. | | | | | | | | |
| 47 | BRC22/0011 | - | ./. | | | | | | | | |

*cBioPortal for Cancer Genomics was used to evaluate the biological effect of the variants

** these variants are not described in common databases (COSMIC, ClinVar, cbioportal.org, JAX CKB, CIVIC, Varsome)

**Supplemental Table S4.** Fluorochrome-conjugated antibodies for cell sorting

| **Cell subtype** | **Antibodies** | **Manufacturer** | **Clone** |
| --- | --- | --- | --- |
| T cells | CD3APC-A700 | Beckman Coulter | UCHT1 |
| B cells | CD19 ECD | Beckman Coulter | J3-119 |
| NK cell | CD56 PE | BioLegend | HCD56 |
| Leucocytes (common antigen) | CD45Kro | Beckman Coulter | J33 |
| Monocytes | CD14 PC7 | Beckman Coulter | RM052 |
| Granulocytes | CD66b PerCP-Cy5.5 | BD Biosciences | G10F5 |

**Supplemental Table S5.** VAFs of CHIP-related mutations identified in flow-sorted

hematopoietic subpopulations

| No | **Gene** | **Exon** | **HGVS.c** | **HGVs.p** | **VAF [%]** | | | | | |
| --- | --- | --- | --- | --- | --- | --- | --- | --- | --- | --- |
|  |  |  |  |  | **WB** | **NK cells** | **Mono-cytes** | **Granulo-cytes** | **T cells** | **B cells** |
| 1 | TET2 | 3 | c.1557dup | p.Gly520TrpfsTer2 | 40 | 43 | 47 | 46 | ./. | 48 |
|  | PHF6 | 10 | c.1030_1031de | p.Ser344Ter | 76 | 82 | 90 | 92 | 3 | 94 |
| 2 | TET2 | 3 | c.1630C>T | p.Arg544Ter | 4 | 2 | 9 | 7 | ./. | ./. |
|  | TET2 | 3 | c.3236dup | p.Ala1080SerfsTer24 | 6 | 11 | 9 | 8 | ./. | 15 |
|  | U2AF1 | 6 | c.470A>G | p.Gln157Arg | 5 | 3 | 10 | 8 | ./. | ./. |
|  | PHF6 | 2 | c.92dup | p.Leu31PhefsTer5 | 40 | 52 | 43 | 47 | 7 | 49 |
| 3 | TET2 | 3 | c.2888del | p.Gln963ArgfsTer44 | 16 | 4 | 21 | 20 | ./. | ./. |
| 4 | TET2 | 3 | c.651del | p.Val218TrpfsTer32 | 3 | 5 | 5 | 4 | ./. | ./. |
| 5 | TET2 | 11 | c.4544del | p.Leu1515CysfsTer5 | 2 | 2 | 5 | 4 | ./. | ./. |
|  | TET2 | 11 | c.5734C>T | p.His1912Tyr | 8 | 10 | 10 | 10 | ./. | 6 |
|  | ASXL1 | 12 | c.1249C>T | p.Arg417Ter | 2 | 4 | 3 | 3 | ./. | 4 |
|  | U2AF1 | 6 | c.470A>G | p.Gln157Arg | 2 | 2 | 5 | 4 | ./. | ./. |

**Supplementary Table S6.** Distribution of bypass grafts

| **Type of peripheral bypass graft** | **Total**  *No (%)* | **Occluded bypass graft** | | | **Open bypass graft** | | |
| --- | --- | --- | --- | --- | --- | --- | --- |
|  |  | **CHIP+**  *No (%)* | | **CHIP-**  *No (%)* | **CHIP+**  *No (%)* | | **CHIP-**  *No (%)* |
| **Number of patients** | 47 | 10 | 23 | | 4 | | 10 |
| **Femoropopliteal** |  |  | | |  | | |
| autogenous greater saphenous vein (GSV) | 10 (21.3) | 2 (20.0) | | 3 (13.0) | 2 (50.0) | | 3 (30.0) |
| prosthesis graft | 27 (57.4) | 5 (50.0) | | 14 (60.9) | 1 (25.0) | | 7 (70.0) |
| **Femorocrural** |  |  | | |  | | |
| autogenous greater saphenous vein | 3 (6.4) | 1 (10.0) | | 2 (8.7) | 0 (0) | | 0 (0) |
| prosthesis graft | 5 (10.6) | 1 (10.0) | | 3 (13.0) | 1 (25.0) | | 0 (0) |
| **Unknown** | 2 (4.3) | 1 (10.0) | 1 (4.3) | |  |  | |

**Supplementary Table S7**. Laboratory data

|  | **Total**  *No or*  *mean ± SD* | **Occluded bypass graft** | | **Open bypass graft** | |
| --- | --- | --- | --- | --- | --- |
|  |  | **CHIP+**  *No or*  *mean ± SD* | **CHIP-**  *No or*  *mean ± SD* | **CHIP+**  *No or*  *mean ± SD* | **CHIP-**  *No or*  *mean ± SD* |
| **Number of Patients** | 47 | 10 | 23 | 4 | 10 |
| **Parameter** |  | | | | |
| WBC (x10^9^/l) | 9.20 *±* 2.64 | 10.24 *±* 2.84 | 9.64 *±* 2.84 | 7.40 *±* 2.53 | 7.89 *±* 1.77 |
| Hemoglobin (g/dl) | 12.80 *±* 3.18 | 11.21 *±* 2.34 | 12.53 *±* 2.86 | 15.58 *±* 1.16 | 13.98 *±* 3.89 |
| Thrombocytes(x10^9^/l) | 243.1 *±* 79.4 | 264.2 *±* 72.2 | 249.1 *±* 96.0 | 233.0 *±* 46.9 | 212.4 *±* 45.3 |
| CRP^1^ (mg/dl) | 2.11 *±* 4.33 | 2.23 *±* 1.77 | 3.14 *±* 5.91 | 0.10 *±* 0.04 | 0.41 *±* 0.35 |
| Creatinine (mg/dl) | 1.09 *±* 0.52 | 1.09 *±* 0.36 | 1.08 *±* 0.68 | 1.17 *±* 0.09 | 0.94 *±* 0.14 |
| Cholesterol (mg/dl) | 153.9 *±* 54.2 | 135.2 *±* 52.5 | 152.1 *±* 44.5 | 196.3 *±* 72.1 | 159.2 *±* 64.4 |
| Missing data | 3 | 1 | 2 |  |  |
| Triglyceride (mg/dl) | 191.2 *±* 336.0 | 119.7 *±* 73.6 | 145.2 *±* 64.8 | 122.8 *±* 21.9 | 379.6 *±* 688.2 |
| Missing data | 3 | 1 | 2 |  |  |
| HDL^2^ (mg/dl) | 50.66 *±* 32.06 | 41.89 *±* 11.35 | 45 *±* 12.12 | 69.0 *±* 46.81 | 63.10 *±* 57.14 |
| Missing data | 3 | 1 | 2 |  |  |
| LDL^3^ (mg/dl) | 90.75 *±* 46.06 | 80 *±* 44.40 | 84.90 *±* 38.38 | 113.0 *±* 53.77 | 103.80 *±* 59.49 |
| Missing data | 3 | 1 | 2 |  |  |
| Lp (a)^4^ (nmol/l) | 94.67 ± 107.50 | 29 ± 70.65 | 81.84 ± 89.56 | 37.67 ± 53.12 | 124.67 ± 164.68 |
| Missing data | 13 | 2 | 7 | 1 | 3 |

^1^CRP - C reactive protein; ^2^HDL - high-density lipoprotein; ^3^LDL - low density lipoprotein; ^4^Lp(a) - lipoprotein (a)

**Supplementary Table S8**. Medication

|  | **Total**  *No (%)* | **Occluded bypass graft** | | **Open bypass graft** | |
| --- | --- | --- | --- | --- | --- |
|  |  | **CHIP+**  *No (%)* | **CHIP-**  *No (%)* | **CHIP+**  *No (%)* | **CHIP-**  *No (%)* |
| **Number of Patients** | 47 | 10 | 23 | 4 | 10 |
| **Antiplatelet therapy** |  |  |  |  |  |
| ASS | 31 (66.0) | 8 (80.0) | 13 (56.5) | 2 (50.0) | 8 (80.0) |
| ASS + Clopidogrel® | 9 (29.0) | 1 (10.0) | 6 (26.0) | 1 (25.0) | 1 (10.0) |
| **Anticoagulation** |  |  |  |  |  |
| Eliquis® | 1 (2.1) | 0 (0) | 0 (0) | 0 (0) | 1 (10.0) |
| Marcumar® | 5 (10.6) | 2 (20.0) | 0 (0) | 1 (25.0) | 2 (20.0) |
| Xarelto® | 5 (10.6) | 1 (10.0) | 4 (17.4) | 0 (0) | 0 (0) |
| Lixiana® | 4 (8.5) | 0 (0) | 1 (4.3) | 0 (0) | 3 (30.0) |
| **Statines** | 35 (74.5) | 8 (80.0) | 16 (70.0) | 3 (75.0) | 8 (80.0) |
| **Anti-hypertensives** | 34 (72.3) | 6 (60.0) | 17 (74.0) | 3 (75.0) | 8 (80.0) |
| **Anti-diabetics** | 17 (36.2) | 4 (40.0) | 9 (39.1) | 2 (50.0) | 2 (20.0) |

**Supplementary Table S9.** Descriptive table of the five *TET2*-mutant patients

| **Number of *TET2* patients** | **Gene** | **VAF** | **Age (years)** | **Sex** | **PAD stage (Fontaine)** | **Conduit type** | **Bypass level** | **Time surgery to graft occlusion (months)** | **Anticoagulation** |
| --- | --- | --- | --- | --- | --- | --- | --- | --- | --- |
| 1 | - TET2; c.1557dupT; p.Gly520TrpfsTer2 | 40% | 77 | male | III | **prosthetic** | **femoropopliteal** | 4 | ASS |
| 2 | - TET2; c.1630C>T; p.Arg544Ter - TET2; c.3236dup; p. Ala1080SerfsTer24 | 4%  6% | 77 | male | III | **prosthetic** | **femoropopliteal** | 23 | ASS |
| 3 | - TET2; c.2888delA; p.Gln963ArgfsTer44 | 16% | 68 | male | II | **prosthetic** | **femoropopliteal** | 9 | ASS |
| 4 | - TET2; c4544del p.Leu1515CysfsTer56 - TET2; c.5734C>T; p.His1912Tyr | 2%  8% | 63 | male | III | **prosthetic** | femorocrural | 38 | ASS+ Xarelto® |
| 5 | - TET2; c651del; pVal218TrpfsTer32 | 3% | 75 | male | IV | unknown | femorocrural | 11 | ASS+ Lixiana® |

In patients with occlusion, blood sampling coincided with presentation of graft occlusion; the interval shown therefore also represents the time from surgery to CHIP assessment.

**Supplementary Table S10**. Regression model and sensitivity analyses

| **Modell** | **Exposure** | **Covariates** | **HR [95% CI]** | **P value** |
| --- | --- | --- | --- | --- |
| Cox | TET2 | smoking, diabetes | 6.70 [1.96; 22.96] | 0.003 |
| Cox | CHIP | smoking, diabetes | 2.79 [0.96; 8.11] | 0.059 |
| Cox | TET2 | age, sex | 4.65 [1.55; 13.90] | 0.006 |
| Cox | CHIP | age, sex | 2.70 [1.05; 6.92] | 0.039 |
| Cox | TET2 | age, smoking | 5.67 [1.66; 19.34] | 0.006 |
| Cox | CHIP | age, smoking | 2.43 [0.89; 6.62] | 0.082 |
| Cox | TET2 | sex, diabetes | 5.11 [1.68; 15.58] | 0.004 |
| Cox | CHIP | sex, diabetes | 3.14 [1.18; 8.34] | 0.021 |
| Cox | TET2 | age, sex, diabetes, smoking | 9.00[1.88; 23.50] | 0.003 |
| Cox | CHIP | age, sex, diabetes, smoking | 2.75 [0.95; 8.01] | 0.063 |
| Cox | TET2 | age, sex, diabetes, smoking, CHD | 8.00 [2.16; 29.61] | 0.002 |
| Cox | CHIP | age, sex, diabetes, smoking, CHD | 2.30 [0.76; 6.94] | 0.139 |
| Cox | TET2 | *PAD stage | 6.13 [1.71;21.67] | 0.005 |
| Cox | CHIP | PAD stage | 2.99[0.97;9.21] | 0.057 |
| Weighted Cox | TET2 | smoking, diabetes | 6.99 [2.06; 23.73] | 0.004 |
| Weighted Cox | CHIP | smoking, diabetes | 2.76 [0.96; 8.00] | 0.057 |
| Weighted Cox | TET2 | age, sex | 5.09 [1.58; 16.45] | 0.012 |
| Weighted Cox | CHIP | age, sex | 2.55 [0.97; 6.70] | 0.058 |
| Weighted Cox | TET2 | age, smoking | 5.94 [1.76; 20.07] | 0.008 |
| Weighted Cox | CHIP | age, smoking | 2.41 [0.89; 6.57] | 0.082 |
| Weighted Cox | TET2 | sex, diabetes | 6.88 [1.97; 24.00] | 0.005 |
| Weighted Cox | CHIP | sex, diabetes | 3.05 [1.11; 8.40] | 0.031 |
| Weighted Cox | TET2 | age, sex, diabetes, smoking | 6.73 [1.90; 23.85] | 0.006 |
| Weighted Cox | CHIP | age, sex, diabetes, smoking | 2.72 [0.94; 7.86] | 0.059 |
| Weighted Cox | TET2 | age, sex, diabetes, smoking, CHD | 7.96 [2.15; 29.52] | 0.003 |
| Weighted Cox | CHIP | age, sex, diabetes, smoking, CHD | 2.24 [0.74; 6.72] | 0.141 |

*PAD stage refers to the Fontaine classification at enrolment, because enrolment stage partly reflects the occlusion event, models adjusted for PAD stage represent a conservative sensitivity analysis.
